# Decentralized Clinical Trials in Pediatrics: Is Italy Ready?

**DOI:** 10.3390/jcm14196906

**Published:** 2025-09-29

**Authors:** Gilda Paternuosto, Anna Flamigni, Monica Zanier, Barbara Bonifacio, Giulia Schillani, Mario Cirino, Sasa Krcalic, Silvia Tommasi, Fatima Tizi, Anna Arbo, Davide Zanon, Alessandra Maestro

**Affiliations:** Institute for Maternal and Child Health, IRCCS Burlo Garofolo, 34137 Trieste, Italy

**Keywords:** clinical trial regulations, decentralized clinical trials, digital health technologies, engagement, informed consent, pediatric clinical trial

## Abstract

Driven by technological advances and regulatory developments, the clinical trials industry is undergoing a period of transformation. Decentralized clinical trials (DCTs) promise to streamline processes, increase participant engagement and improve data quality. The pediatric clinical trial landscape is challenging, as participation in a clinical trial affects the whole family. DCTs allow for increased patient engagement, thereby improving trial quality and consequently drug safety. The Italian Medicines Agency (AIFA), in its quest for regulatory simplification and alignment with the European framework, has established comprehensive guidelines to facilitate these decentralized trials. The present work aims to delve into the regulations to understand the feasibility of conducting decentralized pediatric trials in Italy.

## 1. Introduction

The realm of clinical trials is witnessing a transformative phase, driven by technological advances and regulatory developments. At the heart of this revolution are decentralized clinical trials (DCTs), which promise to streamline processes, increase participant engagement, and improve data quality.

A DCT is a patient-centered clinical trial that leverages new technologies to allow trial-related activities to take place at locations other than traditional trial sites, making clinical trials more accessible and participation more convenient for trial participants [1,2].

The first DCT dates back to 2011, when the REMOTE Phase 4 trial tested a novel web-based trial design (NCT01302938). Web-based methods were used to recruit and screen patients for eligibility, obtain informed consent, and enable the creation of patient e-diaries. The investigational medicinal product (IMP) was mailed directly to participants, and laboratory testing was performed in the patient’s community laboratory. The data showed consistency with results from conventional trials [3], opening new avenues for clinical trials around the world.

With the development of DCTs, digital health technologies (DHTs) have proven essential, enabling remote visits and continuous data collection [1,2]. These technologies have gained momentum especially during the COVID-19 pandemic. Telemedicine, videoconferencing and digital health applications are some examples of how DCTs have improved accessibility and equity in healthcare [4,5]. DHTs proved essential during the pandemic, particularly in the context of clinical trials, as the pandemic led to inevitable protocol deviations [6], and many trials adopted remote patient monitoring to safely continue operations [1,7,8]. These adaptations have accelerated the proliferation of DCTs and fostered close collaboration between sponsors and sites to incorporate these innovative methods [9]. Certainly, trials with DHTs require strong technological support not only for clinical staff, but also for patients and caregivers. In addition, adequate local Internet service is required to ensure that data can be properly transmitted from the patient to the clinical staff.

DCTs can be either hybrid or fully decentralized. Unlike a full DCT, where all activities are remote, a hybrid DCT may be necessary if the trial protocol includes some clinical activities, such as radiological imaging, surgery, and cell or gene therapy, that require occasional on-site patient presence [2].

Trial recruitment is one of the first limiting steps in getting a trial underway. In the pediatric population, recruiting a sufficient number of patients is even more challenging; enrolling a child in a clinical trial inevitably involves the whole family, with parents having to take time off work.

In addition, most diseases affecting children are rare diseases; 70% of the population affected by a rare disease is pediatric, and approximately 95% of these diseases have no approved treatment [10].

Brewster and co-workers [11] analyzed the ClinicalTrials.gov database between 2007 and 2020 and found a high rate of discontinuation of pediatric clinical trials (11.1%), with poor recruitment being the most common reason. Another study shows that barriers such as parental education and socioeconomic status play a critical role in the enrollment of pediatric patients in clinical trials [12]. Delays in recruiting the required sample size can increase costs and disrupt timelines, discouraging sponsors from conducting pediatric clinical trials.

Decentralized trials have shown an increase in the retention rate from 60% in traditional clinical trials to 89% in DCTs [13]. In clinical trials involving pediatric patients, DCTs are undoubtedly convenient for families, allowing the study to be conducted mainly at home, with less burden for both children and parents. Moreover, the continuous collection of real-world data through wearables and telemedicine practices increases both the quality and quantity of data collected [13,14].

Since the REMOTE clinical trial, regulatory agencies in several countries have developed frameworks to guide the design and implementation of DCTs [1]. An EU DCT project, coordinated by the Clinical Trials Coordination Group (CTCG), is harmonizing the use of DCT elements in clinical trials. Some DCT elements are still under development in many Member States and require further guidance and harmonization in the EU, such as:

- Teleconsultation or home visits by health professionals to study participants’ homes;
- Direct shipment of IMPs to trial participants;
- Electronic informed consent processes [15].

To simplify regulations and align with the European framework, the Italian Medicines Agency (AIFA) created a document with guidelines to facilitate these decentralized trials [16]. The guidelines cover topics like:

- Use of third-party service providers,
- Expense reimbursement for clinical trial participants,
- Compensation for loss of earnings for clinical trial participants,
- Delivery of investigational product to participant’s home,
- Allocation of experimental and ancillary drug costs,
- Clinical trials in non-hospital settings.

When analyzing the European regulation and the Italian guideline with respect to the pediatric population, a number of considerations must be taken into account. The ensuing discussion section methodically addresses these considerations.

## 2. Discussion

A thorough search of the ClinicalTrials.gov database yielded a total of three studies relevant to the search term “decentralized” and the location “Italy,” all of which are observational in nature: NCT06941025, NCT05550961, and NCT06337578.

The objective of the study, NCT06941025, is to collect data from women and their infants using a secure web-based platform. This platform enables patients and physicians to input information via electronic case report forms (eCRFs), thereby facilitating remote participation and data management.

Study NCT05550961 aims to establish a national lung cancer network by developing a decentralized, long-term database and a virtual, multi-tiered biobank. However, in this context, decentralization refers to the structural design of data storage and resource sharing, rather than to the operational methodology of the trial itself.

The objective of the study NCT06337578 is the development of advanced telephone-based cognitive screening procedures for adult patients. This approach suggests a shift toward the utilization of remote assessment tools, while maintaining an observational framework.

In order to comprehend the absence of DCTs in Italy, particularly within the pediatric context, it is essential to consider several factors. As illustrated in Table 1, the Italian regulatory framework concerning DCTs is examined, with a focus on the identified regulatory gaps and the proposed solutions to address these gaps. These proposed solutions are subsequently discussed in detail.

The implementation of DCTs, particularly in a pediatric context, addresses numerous challenges for all the stakeholders involved. It is imperative that sponsors allocate resources toward the acquisition of dependable technological systems. Investigators and families must undergo training on the utilization of tools that are subject to constant evolution. Ethics Committee members are required to develop familiarity with novel technologies, their potential, and their associated risks, in order to facilitate an adequate evaluation of the trial protocol.

### 2.1. Electronic Recruitment

As previously mentioned, trial recruitment is one of the first limiting steps in the initiation of a trial. When it comes to the pediatric population, recruiting a sufficient number of patients is even more challenging because of the prevalence of rare diseases and the involvement of the entire family.

The issue of efficiency in clinical research enrolment has long been a concern of AIFA [17]. The improvement and facilitation of access to clinical research through electronic recruitment is an important element in the implementation of DCTs, as it allows the patient to increasingly become a partner of choice.

The Web and social networks are highly flexible and widely available tools. By facilitating the connection between the site and the patient, digital enrollment can offer the advantage of improving overall enrollment. Web-based services, particularly e-recruitment, are proposed as a valuable tool and ally for research staff, facilitating remote activities in conducting patient enrollment in clinical trial programs and creating new connections between patients and research centers [17]. A review based on online randomized trials in children concluded that social media and other online advertising accelerated recruitment efforts [18].

So far, eRecruitment in Italy is very limited, with only three companies authorized in 2020–2021, probably because the ethics committee requires a full review of the website used for eRecruitment so that a Data Protection Impact Assessment (DPIA) can be conducted as required by Regulation (EU) 679/2016 and cyber security can be ensured [17]. With the exception of the Decree of 21 December 2007 [19], which regulates recruitment methods in terms of possible and forbidden promotional aspects, there is no relevant legislation regulating eRecruitment in Italy.

The 2022 National Institute of Health report [17] highlights how the lack of national clinical trials information in Italy can hinder eRecruiting, as it is difficult for patients and carers to easily access information on clinical trials that are currently recruiting. In this regard, the European Medicines Agency has just launched (in March 2025) a new clinical trial map accessible from the Clinical Trials Information Systems (CTIS) to allow patients and healthcare professionals to easily access information on the ongoing clinical trials [20].

The implementation of national guidelines and a standard DPIA template for eRecruitment platforms has the potential to enhance the recruitment process in pediatric clinical trials.

### 2.2. Informed Consent

The informed consent process consists of two steps. In the first step, the physician explains the contents of the consent form to the patient and is available to answer any questions the patient may have about any aspect of the study. If the patient agrees, the second step is to date and sign the consent form.

The Italian national guidelines for the provision of telemedicine services allow the use of SMS, encrypted e-mail and video as differentiated tools for communicating with the patient, also with the help of territorial facilities close to the patient’s home [21]. In a DCT, patients will be able to examine the study documents in a digital format from the comfort of their own home. The material can be sent by e-mail or through a website with a personal login and is supplemented by a video call with the principal investigator of the study [17].

The new ICH E6(R3) Annex 2 recognizes that informed consent may be obtained remotely and states that it should be tailored to the trial design. If obtained remotely, the investigator should verify the identity of the participant or the participant’s legal representative. The informed consent should describe the type of data that will be collected, how the data may be used, and who will have access to the patient’s data [22].

The requirements for informed consent in pediatric clinical trials vary across Europe. Each European country has its own age range for consent that is legally required or suggested. The number of required signatories also varies by country [23]. In Italy, the legal framework governing clinical trials requires the signature of both living parents or legal representatives on consent forms for child participants. Furthermore, for children who have reached a minimum age of six years, it is obligatory to obtain assent from them for their inclusion in the trial.

Information strategies must be adapted according to age groups. Specifically, there is a need for distinction between the communication tools and processes employed with children under 11 and those employed with “mature minors” aged 12 to 17. However, chronological age alone is not a sufficient criterion for assessing maturity. The onus falls upon the investigator to evaluate each minor’s cognitive and emotional development on an individual basis prior to proposing their participation in a trial [24].

Ensuring that the child comprehends the study’s purpose, potential outcomes, and their rights can pose significant challenges during the evaluation of a remote study. The use of video, pictures, and interactive elements can reduce protocol deviations and improve data quality. Nonetheless, it is imperative to acknowledge that electronic instruments must not supplant the interaction between patient and investigator. This interaction remains paramount for children to acquire the requisite information necessary to provide or withhold their final assent. Therefore, a remote connection between the trial participant’s home and the trial site is to be established.

In accordance with the provisions outlined in Regulation (EU) 910/2014 (eIDAS, electronic IDentification, Authentication and trust Services), the utilization of digital signatures within the context of the informed consent process is permitted, contingent upon the fulfillment of the stipulated criteria for authenticity, integrity, and legal validity [25]. In the Italian context, Legislative Decree No. 82/2005 [26], as amended, must also be considered. Article 3 of the Decree formally acknowledges the right of individuals to utilize technological tools in an accessible and effective manner, including for the purpose of participating in administrative procedures. Additionally, the Decree of the President of the Council of Ministers dated February 22, 2013, delineates the technical specifications for the creation, implementation, and verification of advanced, qualified, and digital electronic signatures, as well as the temporal validation of such signatures and the roles of accredited certifiers [27].

Despite the existence of a regulatory framework, the AIFA guidelines on decentralized clinical trials do not provide specific guidance regarding the types of electronic signature tools or platforms that are considered acceptable or compliant within the Italian healthcare and research system. This regulatory lacuna engenders uncertainty for sponsors and investigators, particularly with regard to compliance with the General Data Protection Regulation (GDPR) and the secure handling of sensitive health data in remote consent procedures.

Privacy risks related to platform vulnerabilities and the potential for data breaches must be duly considered [28]. Moreover, loss of metadata may result in incomplete traceability of the consent process and may lead to audit challenges [29]. Building a national registry of validated eConsent platforms may help overcome privacy risks.

In practice, it is imperative that the digital signature employed in clinical research is legally valid, incorporates the signatory’s identity, and ensures the authenticity, integrity, and non-repudiation of the consent. Furthermore, the execution of clinical trials frequently necessitates a paper-based version of the consent form or the implementation of additional verification steps to ensure traceability. Therefore, it is advisable to confirm the specific requirements of both the sponsor and the regulatory authority to ensure full compliance with applicable standards and expectations.

In a recent opinion on a study protocol, the local ethics committee of the Friuli Venezia Giulia region (north-east Italy) has clarified that, while the possibility of obtaining consent remotely is potentially feasible, direct contact with participants is certainly preferable. Therefore, in the context of obtaining consent remotely, it is imperative to provide a rationale that justifies this choice. In addition, the participating centers are required to indicate whether they possess specific procedures for this method of obtaining informed consent/refusal—consent/nonconsent. These procedures must be verified as feasible in advance.

### 2.3. Use of Third-Party Services

The involvement of third parties may be deemed essential in the context of DCTs. According to the provisions stipulated in Regulation (EU) 679/2016, the term “third party” is defined as “a natural or legal person, public authority, agency, or body other than the data subject, the controller, the processor, and persons who, under the direct authority of the controller or processor, are authorized to process personal data” [30].

In the case of a DCT, the delivery of the IMP to the participant’s home or the provision of a home nurse is typically delegated to a service provider. The utilization of these services must be explicitly delineated in the protocol, in accordance with good clinical practice as delineated in ICH E6(R3), and the service provider must be duly informed about the study protocol [16]. The European GDPR is a comprehensive set of data protection regulations that service providers and their employees must adhere to when processing individuals’ data [30]. It is imperative that a contract elucidate the roles concerning data protection and security prior to the initiation of a study [16], and that this contract be extensively reviewed by the Ethics Committee

In instances where the clinical trial encompasses a pediatric population, the GDPR serves to reinforce the regulatory framework concerning data protection. Children are particularly vulnerable to the processing of their personal data due to their limited understanding of the risks, consequences, protections, and rights associated with the processing of their personal data. Specific legal provisions are required that pertain to the utilization of services that are directed at the child, and all forms of information and communications directed at a minor must be articulated in a manner that is easily comprehensible to them [30].

### 2.4. Expense Reimbursement for Clinical Trial Participants

The reimbursement of expenses related to the participant’s site visit, including but not limited to food, lodging, and transportation, is contingent upon approval by the Ethics Committee. Given the legal requirement that minors be accompanied by their parents or legal guardians, it is crucial to consider the reimbursement of expenses incurred by the accompanying individual.

Reimbursement may be provided by a third party. The guidelines [16] underscore the significance of meticulously documenting the role of a third party in the contractual agreement between the trial site and the sponsor, as well as in the trial protocol.

The AIFA guidelines lack clarity regarding the roles of the various actors involved; however, the recommendations provided by the Territorial Ethics Committees [31] delineate specific requirements. These include a contract between the service provider and the trial site, subsequent to adequate verification of the service provider’s competence and independence by the trial site. The contract must explicitly state that the service provider functions as a data processor and that the trial center is responsible for remunerating the service provider for its role in managing the reimbursement of expenses, utilizing funds provided by the sponsor.

It is noteworthy that DCTs can greatly reduce the cost of the study, as the study is mainly conducted at the participant’s home, thus limiting the number of site visits.

### 2.5. Compensation for Loss of Earnings for Clinical Trial Participants

Compensation for loss of earnings is intended for healthy volunteers participating in Phase 1 clinical trials [16]. As the enrollment criteria for pediatric clinical trials exclusively include patients, this particular is not applicable to pediatric trials [32].

### 2.6. Delivery of the Investigational Product to the Participant’s Home

The AIFA Guideline on Regulatory Simplification and Decentralization Elements has identified a hospital pharmacy, a delegated territorial pharmacy, a warehouse, or a service provider as potential delivery services for IMPs [16].

Drugs that may be suitable for direct delivery to the patient’s home are those that have a favorable stability profile, minimal risk of hypersensitivity or potential abuse, minimal handling requirements (e.g., no need for extemporaneous preparation of the IMP in a sterile environment), and a known safety profile. Examples of suitable drugs include pediatric drugs that have already been tested for safety in adults or drugs approved for other indications that have detailed safety information regarding adverse reactions and toxicity management.

In addition, home dosing regimens should be meticulously planned in advance, as evidenced by dose escalation studies. These studies underscore the intricacies inherent in home medication management and the indispensable role of caregivers [33]. In this context, the potential for IMPs to be supplied by a delegated, GCP-certified community pharmacy could play a significant role in reducing the burden on caregivers, despite the absence of explicit regulations in this regard [34].

In accordance with the protocol of a conventional clinical trial, the decentralized protocol is required to adhere to the Good Distribution Practice (GDP) standards [35].

The hospital pharmacist is responsible for ensuring the maintenance of the physical integrity and stability of the IMPs. This responsibility encompasses the methods of IMP packaging control, shipment tracking, and temperature monitoring by means of temperature tracking devices.

Furthermore, the rigorous procedures for tracking the receipt of the medicinal product and for returning any leftover product to the sponsor for disposal that are required for clinical trials [22] are also in place. It is imperative that these procedures be delineated in the trial protocol, irrespective of the involvement of decentralized sites.

The hospital pharmacist functions in a direct capacity with the third-party service, overseeing the adherence to regulatory standards for storage and quality of home deliveries. This ensures stability, drug integrity, delivery timing, and transport documentation. Furthermore, the pharmacist will furnish pertinent and superior data to investigators by means of monitoring medication adherence, calculating remnants, and tracking batches through data collection and recording in electronic platforms. The pharmacist will also educate patients and caregivers about pharmacovigilance and device vigilance. This encompasses the reporting of suspected adverse reactions observed at home by the patient or caregiver.

In this manner, the hospital pharmacist is responsible for ensuring the maintenance of the quality and efficacy of IMPs. To this end, the pharmacist must guarantee that all processes related to investigational medicinal products comply with the regulatory requirements of EU Regulation 536/2014 [36]. These processes include distribution and administration. The efficacy of a decentralized trial is contingent upon the effective management of the IMP, a critical component that warrants meticulous attention to ensure its optimal functioning. The employment of validated delivery partners and the assurance of pharmacist oversight within the study protocol may enhance the efficacy of DCTs.

### 2.7. Data Collection

The National Center for Telemedicine and New Health Technologies of the Italian National Institute of Health and Farmindustria investigated innovative methodologies for conducting DCTs in Italy by disseminating real-world data (RWD) and establishing collaborative clinical research networks leveraging digital technologies [17].

The utilization of digital tools facilitates the aggregation of substantial quantities of high-fidelity RWD, thereby enhancing patient compliance. The aforementioned instruments can be categorized as follows:

- Remote monitoring systems that facilitate remote sensing and continuous transmission of vital and clinical parameters through patient-interacting sensors (biomedical technologies with or without applied parts) [17,21,37].
- Remote visits that can be utilized for follow-up purposes [17,21,37].
- Remote support, such as trial information and patient education via videos or alerts for IMP administration via messages, applications, reminders, and eDiaries [17].
- Electronic Patient Reported Outcomes (ePROs) and Electronic Clinical Outcome Assessments (eCOAs) to collect clinical data. ePROs refer to outcome measures as reported by patients in electronic forms, whereas eCOAs are outcome measures collected through technologies such as electronic devices, tablets or websites by both patients and healthcare providers [17].
- Patient experience and quality of life surveys [17].

The efficacy of ePROs in engaging children, irrespective of age, in their healthcare has been demonstrated. The integration of these systems into the daily lives of children has the potential to engage them without the encumbrance of conventional data collection methodologies [38].

The implementation of technological systems, including wearables, mobile applications, and telehealth platforms, necessitates adaptation to the specific needs of the target age group. Caregiver training may be imperative to ensure the proper usage of DHTs and the completeness of the collected data. Furthermore, the design of pediatric DHTs must consider the anatomical and physiological changes associated with the child’s growth during the clinical trial [39].

The utilization of DHTs and remote monitoring carries potential risks to privacy and confidentiality. This is due to the possibility of disclosure or breach of data collected, transmitted, and stored via digital platforms. Consequently, there is an increased need for security measures to protect data from breaches during collection, transmission, and storage. The AIFA Guidelines do not include explicit requirements for data collection systems or the validation of these systems. However, distributed ledgers (e.g., blockchain or decentralized databases) have the potential to mitigate the risk of database breaches [28,40].

The management of data is inextricably linked to the principles outlined in the GDPR. According to Art. 32 of the GDPR, sponsors and promoters are obligated to implement advanced cybersecurity systems and procedures for the purpose of reporting data breaches within 72 h. To ensure operational transparency and compliance, records of processing activities and periodic audits must be maintained.

## 3. Conclusions

Italy currently faces a critical juncture in the progression of DCTs, particularly in the context of the pediatric population. While the potential benefits of DCTs—such as increased accessibility, reduced logistical burdens, and enhanced patient engagement—are evident, the current regulatory and infrastructural landscape reveals significant gaps that must be addressed to ensure readiness and efficacy.

Notwithstanding the existence of overarching frameworks, including AIFA guidelines and EU regulations, critical ambiguities persist. The dearth of explicit directives pertaining to electronic signature platforms, remote consent procedures, and the roles of third-party services engenders a pervasive sense of uncertainty among sponsors and investigators, particularly with respect to navigating the intricate landscape of GDPR compliance and the safeguarding of sensitive pediatric health data. The distinct vulnerability of children in data processing underscores the necessity for customized legal provisions and communication strategies that prioritize clarity and comprehension. It is encouraging to note that Italy has initiated the exploration of innovative methodologies through national initiatives, leveraging DHTs such as remote monitoring, ePROs, and telehealth platforms. The efficacy of these tools in promoting pediatric engagement and generating high-fidelity RWD has been demonstrated. However, the successful integration of these technologies is contingent upon the implementation of age-appropriate design, the provision of caregiver training, and the capacity to adapt to the physiological changes that are characteristic of pediatric development. Furthermore, individuals residing in rural areas or socioeconomically disadvantaged settings may encounter impediments to participation due to their limited digital literacy and connectivity. This situation has the potential to exacerbate existing disparities in health.

Operational and ethical challenges persist. The absence of comprehensive traceability in consent and data flows has the potential to impede auditability and undermine the credibility of trials. The absence of standardized documentation protocols and interoperable systems has been demonstrated to have a significant impact on the complexity of regulatory compliance. Local Ethics Committees frequently exhibit a paucity of harmonized criteria for evaluating digital tools, remote procedures, and third-party involvement. This deficiency often results in potential delays and inconsistencies in participant protection.

In pediatric DCTs, caregivers assume a pivotal role in medication, IMP administration, and data monitoring. In the absence of adequate support and training, there is a risk that this burden may compromise the quality of data, the ethical standards of care and participation.

Prior to the establishment of the AIFA guideline, Italy lacked a formal regulatory framework for DCTs. At this juncture, approximately one year later, and taking into account the time necessary to initiate and conduct studies, there remains a paucity of data on DCTs that have been effectively implemented.

Although Italy has taken preliminary steps toward the adoption of decentralized trials, the establishment of a comprehensive and harmonized regulatory framework—coupled with infrastructural investment and stakeholder education—is imperative, particularly within the area of pediatric research. The realization of the full potential of DCTs to transform pediatric clinical research into a more inclusive, efficient, and patient-centered endeavor is contingent upon the concerted efforts of all the stakeholders involved.

## Figures and Tables

**Table 1:** Considerations on the regulatory framework on DCTs.

	Regulatory Ref	Regulatory Gaps	Proposed Solutions
**eRecruitment**	Reg. (EU) 679/2016 (DPIA); GDPR	No relevant regulation in Italy	Defining national guidelines and DPIA template
**Informed Consent**	Reg. (EU) 910/2014 (eIDAS); D.lg. 82/2005; ICH E6(R3); GDPR	No specific regulation or guidelines on types of e-signature tools or platforms in Italy	Building a national registry of validated eConsent platforms in accordance with national authorities
**Third Party Services**	Reg. (EU) 679/2016; ICH E6(R3); GDPR	--	--
**Reimbursement**	Reg. (EU) 679/2016	AIFA guidelines unclear on role of third parties	Use of GDPR-compliant service providers, Ethics Committee review of third-party contracts
**IMP Delivery**	GDP Guidelines; Reg. (EU) 536/2014	No specific regulation on the role of community pharmacists	Create a register of GCP-certified community pharmacies
**Data Collection**	GDPR	No specific requirements in AIFA guidelines	Use of blockchain databases

Abbreviations: Reg. = Regulation; DPIA = Data Protection Impact Assessment; GDPR = General Data Protection Regulation; eIDAS electronic IDentification, Authentication and trust Services; D.lg = Decree law; ICH = International Council for Harmonisation; AIFA = The Italian Medicines Agency; GDP = Good Distribution Practice; GCP = Good Clinical Practice.

## Abbreviations

The following abbreviations are used in this manuscript:

AIFA	Agenzia Italiana del Farmaco (Italian Medicines Agency)
CTCG	Clinical Trials Coordination Group
CTIS	Clinical Trials Information System
DCT	Decentralized Clinical Trial
DHT	Digital Health Technology
D.lg.	Decree Law
DPIA	Data Protection Impact Assessment
eIDAS	electronic IDentification, Authentication and trust Services
GCP	Good Clinical Practices
GDP	Good Distribution Practices
GDPR	General Data Protection Regulation
ICH	International Council for Harmonisation
IMP	Investigational Medicinal Product
Reg.	Regulation
